# Mortality among transgender persons: a Taiwan matched-population and sibling-comparison cohort study

**DOI:** 10.1016/j.lanwpc.2025.101715

**Published:** 2025-10-18

**Authors:** Chih-Wei Hsu, Yang-Chieh Brian Chen, Liang-Jen Wang, Mu-Hong Chen, Yao-Hsu Yang, Chih-Sung Liang, Po-Yen Chen, Edward Chia-Cheng Lai

**Affiliations:** a Department of Psychiatry, Kaohsiung Chang Gung Memorial Hospital and Chang Gung University College of Medicine, Kaohsiung, Taiwan; b Department of Psychiatry and Behavioral Sciences, The University of Texas Health Science Center at Houston, Houston, TX, USA; c Department of Child and Adolescent Psychiatry, Kaohsiung Chang Gung Memorial Hospital, Chang Gung University College of Medicine, Kaohsiung, Taiwan; d Department of Psychiatry, Taipei Veterans General Hospital, Taipei, Taiwan; e Department of Psychiatry, College of Medicine, National Yang Ming Chiao Tung University, Taipei, Taiwan; f Department of Traditional Chinese Medicine, Chiayi Chang Gung Memorial Hospital, Chiayi, Taiwan; g Health Information and Epidemiology Laboratory of Chang Gung Memorial Hospital, Chiayi, Taiwan; h School of Traditional Chinese Medicine, College of Medicine, Chang Gung University, Taoyuan, Taiwan; i Department of Psychiatry, Beitou Branch, Tri-Service General Hospital, National Defense Medical University, Taipei, Taiwan; j Department of Psychiatry, National Defense Medical University, Taipei, Taiwan; k Department of Urology, Kaohsiung Chang Gung Memorial Hospital, Chang Gung University College of Medicine, Kaohsiung, Taiwan; l Center for Shock Wave Medicine and Tissue Engineering, Kaohsiung Chang Gung Memorial Hospital, Chang Gung University College of Medicine, Kaohsiung, Taiwan; m Graduate School of Human Sexuality, Shu-Te University, Kaohsiung, Taiwan; n School of Pharmacy, Institute of Clinical Pharmacy and Pharmaceutical Sciences, College of Medicine, National Cheng Kung University, Tainan, Taiwan; o Population Health Data Center, National Cheng Kung University, Tainan, Taiwan

**Keywords:** Accidents, Death, Gender dysphoria, Suicide, Transsexualism

## Abstract

**Background:**

Transgender persons have been reported to experience excess mortality, but evidence is dominated by Western cohorts and seldom addresses familial confounding.

**Methods:**

We conducted a nationwide, retrospective cohort study using two prespecified comparison designs: matched population controls (1:4, matched on legal sex and birth date) and within-family cisgender sibling comparisons. Transgender persons were identified by ≥2 psychiatrist-recorded gender identity disorder diagnoses during 2001–2021; cohort entry required age ≥6 years. Participants were followed until death or December 31, 2022. We compared all-cause, external-cause (suicide and accidents), and internal-cause mortality using Cox regression adjusted for sociodemographics and medical comorbidity.

**Findings:**

Among 3906 transgender persons (mean age 24.6 years; 70.6% with a male legal sex) and 15,624 controls (mean follow-up 9.5 years), psychiatric conditions were markedly over-represented in the transgender cohort (e.g., depressive disorders 40.8% vs 5.7%). Adjusted hazard ratios were 1.40 (95% confidence interval 1.06–1.84) for all-cause mortality, 2.03 (1.39–2.96) for external causes, and 4.07 (2.52–6.59) for suicides; accidents (0.56, 0.21–1.45) and internal-cause (0.98, 0.65–1.48) mortality did not differ. In sibling comparisons (4765 siblings), excess all-cause and external-cause mortality were not observed (0.96, 0.67–1.36; and 1.14, 0.70–1.85), suicide risk was higher (2.57, 1.32–5.00), and accident mortality lower (0.19, 0.06–0.67). Relative hazards were greatest in those with a female legal sex and in adolescents. Adjustment for psychiatric clusters attenuated—but did not eliminate—the suicide mortality excess.

**Interpretation:**

In Taiwan, transgender persons had higher all-cause and suicide mortality than population controls; in within-family comparisons, the suicide mortality excess persisted, indicating influences beyond shared familial factors. Prevention should prioritize targeted mental-health and safety interventions, with particular attention to adolescents and those with a female legal sex.

**Funding:**

Taiwan National Science and Technology Council.

**Research in context**

**Evidence before this study**

We searched PubMed for “(transgender OR gender dysphoria OR gender identity disorder) AND mortality” without language restrictions. Registry cohorts from Sweden, the Netherlands, Denmark, Finland, and England consistently report 1.5–3.0-fold excess all-cause mortality and even higher suicide mortality in transgender and gender diverse persons. Findings for accidents and internal causes are mixed, and few studies have explored them. Crucially, nearly all prior investigations compared transgender people with unrelated cisgender population controls, with no within-family designs investigating mortality.

**Added value of this study**

Using an insurance database of Taiwan's population, we assembled a cohort larger than any previous Asian study and uniquely incorporated a cisgender sibling-comparison design. This approach allowed simultaneous estimation of population-level risk and evaluation of unmeasured familial confounding. In population comparisons, transgender persons had higher all-cause, external-cause, and suicide mortality than matched controls. In sibling comparisons, differences in all-cause and external-cause mortality were not observed, whereas suicide mortality remained higher and accident mortality was lower for transgender persons. Adolescents and persons with a female legal sex showed the greatest relative hazards. Across six psychiatric comorbidity clusters, elevated suicidality persisted in most strata, with smaller differences among persons with depressive disorders.

**Implications of all the available evidence**

Across population and within-family designs, elevated suicide mortality in transgender persons is consistent. Practice and policy should prioritize routine, structured assessment and response to suicidality, adolescent-focused support, support for persons with a female legal sex, family-acceptance programs, anti-bullying enforcement, and equitable access to gender-affirming care.

## Introduction

Transgender persons—a diverse population whose gender identity differs from their sex assigned at birth—may present in the healthcare system through a documented diagnosis of gender identity disorder (GID), termed gender dysphoria in the Diagnostic and Statistical Manual of Mental Disorders, Fifth Edition.^1^^,^^2^ Once considered rare, its documented prevalence is increasing.^3^ A systematic review of Western surveys reported 2.6 and 6.8 cases per 100,000 among people assigned female and male at birth, respectively.^3^ Meanwhile, Asian figures vary markedly. Singapore's population registers list 12 and 35 per 100,000 (based on psychiatrist diagnoses).^4^ A Japanese population survey yields point prevalences of 270 and 350 per 100,000 (measured via the Utrecht Gender Dysphoria Scale questionnaire).^5^ Taiwanese insurance data show the one-year prevalence of 3.2 and 7.4 per 100,000.^6^

Research using registry data has shown that transgender persons have an excess risk of mortality—particularly from all causes and suicide—compared with cisgender persons. A nationwide Swedish cohort reported all-cause mortality approximately three times higher and suicide mortality nearly nineteen times higher,^7^ while cohorts from the Netherlands and Denmark similarly found elevated deaths—especially among transfeminine individuals—from suicide, substance misuse, and, to varying degrees, cardiovascular disease or cancer.^8^^,^^9^ These investigations, however, have notable limitations. Few analyze accidental deaths separately, leaving it uncertain whether an excess exists in this domain. Most compare transgender persons with unrelated population controls, so it is unclear whether mortality risks persist when cisgender siblings are used to control for shared familial factors. Moreover, because GID commonly co-occurs with psychiatric diagnoses such as depression,^10^^,^^11^ it remains unknown how risk estimates change after adjusting for specific comorbidities. Finally, large-scale mortality studies in Asia are still lacking in the literature,^12^ underscoring the need for region-specific evidence.

To address these gaps, we aimed to examine all-cause, external-cause (suicide and accidental), and internal-cause mortality among transgender persons in Taiwan, comparing them with matched population controls and cisgender siblings. We further assessed whether mortality patterns varied by legal sex, age, and psychiatric comorbidities.

## Methods

### Data sources

This population-based, retrospective cohort study was conducted in strict accordance with the STrengthening the Reporting of OBservational studies in Epidemiology (STROBE) guidelines (Appendix). Ethical clearance was granted by the Institutional Review Board of Chang Gung Memorial Hospital (approval No. 202300262B0). Because the underlying data had been fully anonymized before release, the requirement for individual informed consent was waived.

All variables were retrieved from the Taiwan National Health Insurance Research Database (NHIRD). Established in 1996 to support public health research, the NHIRD captures essentially every reimbursed outpatient and inpatient encounter for more than 99% of Taiwan's 23 million residents.^13^ For each beneficiary, encrypted personal identifiers permit deterministic linkage across claims, pharmacy dispensations, and the national death registry while preserving confidentiality. The database provides rich demographic information (legal sex, date of birth, income-indexed insurance premium, residential township, and family relations) as well as diagnostic codes for all clinical contacts. Data spanning January 1, 2000, to December 31, 2022, was available for the present investigation. During the study window, Taiwan changed its coding system from the International Classification of Diseases, Ninth Revision, Clinical Modification (ICD-9-CM) to the Tenth Revision (ICD-10-CM) on January 1, 2016. Previous observational studies have demonstrated the robustness and validity of using the NHIRD for epidemiological studies, and several previous studies have used this repository to investigate long-term outcomes in different patient populations.^13^

### Matched and sibling cohorts

We used two complementary comparison designs to address distinct inferential aims: a matched-population cohort to estimate population-level associations, and a within-family sibling comparison to reduce confounding by shared familial factors (e.g., genetic liability and early-life environment). The transgender cohort comprised individuals aged ≥ six years who received at least two psychiatrist-recorded diagnoses of GID (ICD-9-CM 302.5, 302.6, or 302.85; ICD-10-CM F64) on separate visit dates between January 1, 2001, and December 31, 2021. We chose the ≥6-year cutoff based on the typical onset (ages 5–7) of gender dysphoria^14^ and Taiwan's entry into formal schooling at age six, when children begin formal schooling and interact regularly with peers; requiring two diagnoses improves accuracy and aligns with Taiwanese administrative practice whereby eligibility for gender-affirming surgery typically requires independent evaluations by two psychiatrists.^15^ The earliest qualifying encounter defined the index date. For the matched-population design, four comparison participants without any GID diagnosis in the entire NHIRD were randomly selected for each case; controls were individually matched on legal sex and date of birth (within a six-month window) and inherited their case's index date to ensure synchronous follow-up. The earliest recorded sex in the NHIRD (available from 2001 onward), corresponding to the administrative gender marker documented in the insurance claims database, was considered the individual's legal sex. We additionally assembled a sibling cohort for the within-family comparison. In families where at least one child met the GID definition, all non-transgender siblings listed in the insurance file (i.e., individuals sharing at least one parent) were included.

### Follow-up and outcomes

For matched controls, the index date was anchored to the date of the first confirmed GID diagnosis in the corresponding case. For sibling controls, the index date was the earliest of January 1, 2001 (start of NHIRD coverage) or the individual's date of birth if born after that date. Participants were followed from the index date until death or December 31, 2022, whichever occurred first. The primary outcome was all-cause mortality; secondary outcomes were certified underlying causes of death, grouped as external or internal, with the former further subdivided into accidental deaths and suicide.

### Covariates

At the index date we recorded each participant's age, legal sex, and the calendar year of cohort entry. The National Health Insurance premium—calculated from wage-linked salary brackets—is a well-validated surrogate for personal socioeconomic position; we therefore divided monthly premiums into annual quartiles (highest, upper-middle, lower-middle, lowest) derived from the income distribution of the matched controls, an approach that has been shown to capture social gradients in health and health-care use within the Taiwanese universal-coverage system.^16^ Residential context was measured with the Ministry of the Interior urbanization index, which amalgamates five township-level indicators—population density, age structure, proportion of agricultural workers, prevalence of tertiary education, and physician density—and stratifies all townships into hierarchical levels; we stratified urbanization levels into four levels (level one = mostly urbanized, level four = mostly rural), which is consistent with previous NHIRD studies.^17^ We selected these individual characteristics based on prior evidence linking them to mortality risk.^13^^,^^18^

Comorbidities were ascertained from the full Taiwan National Health Insurance claims history and treated as proxies for lifetime diagnoses. Physical health burden was summarized with the Charlson Comorbidity Index (CCI) using 19 chronic medical conditions (myocardial infarction, heart failure, peripheral or cerebral vascular disease, dementia, pulmonary disease, connective tissue disease, peptic ulcer disease, liver disease, diabetes, hemiplegia or paraplegia, renal disease, cancer, and human immunodeficiency virus disease), demonstrating excellent discrimination for long-term mortality using the NHIRD.^19^^,^^20^ Psychiatric comorbidity was classified a priori into six mutually exclusive clusters—neurodevelopmental disorders (intellectual disability, autism spectrum disorder, attention-deficit/hyperactivity disorder, Tourette syndrome, and chronic tic disorder), psychotic disorders, bipolar disorders, depressive disorders, anxiety disorders, and substance use disorders—as each group has been linked to elevated mortality risk.^21^, ^22^, ^23^, ^24^, ^25^, ^26^ These clusters are distinct by Diagnostic and Statistical Manual of Mental Disorders/ICD nosology; however, individuals may have diagnoses spanning two or more clusters. Missing information on income or urbanization was rare; to avoid selection bias, these instances were coded as an “unknown” category and retained in the analysis set. In the NHIRD, to ensure confidentiality and minimize potential identification of individuals, data were only reported if the total number exceeded three within any given category. A comprehensive list of corresponding ICD diagnosis codes and a study flow chart are given in Table S1 and Figure S1, respectively.

### Statistical analysis

Baseline characteristics were summarized separately for each cohort, with continuous variables reported as means ± standard deviations and categorical variables as counts with percentages. Cumulative survival was depicted with Kaplan–Meier curves. Mortality risk was quantified with Cox proportional-hazards models that used time since cohort entry as the underlying time scale; results are presented as hazard ratios (HRs) with 95% confidence intervals (CIs). All Cox models used cluster-robust standard errors—clustered on matched set in the population-matched analyses and on family in the sibling analyses—to account for within-cluster correlation.^27^ Analyses were first performed for all-cause mortality and then repeated for each specific cause of death. Model 1 yielded crude/unadjusted HRs, whereas model 2 adjusted for birth year, sex, income quartile, urbanization level, and CCI.

Two sensitivity analyses were conducted within the matched cohort. First, to avoid bias introduced by the COVID-19 pandemic, we excluded data occurring on or after January 1, 2020. Second, a complete-case analysis was performed in which participants with missing covariate data were excluded. Moreover, to further address shared familial factors, we analyzed a family-clustered cohort including all transgender persons and any siblings (including transgender siblings); index individuals without eligible siblings contributed as singleton clusters. Mortality was compared by transgender status within families using model 1 (unadjusted) and model 3 (adjusted for birth year, legal sex, income quartile, urbanization level, CCI, and birth order). Three subgroup analyses were also applied to the matched cohort. Effect modification was assessed by stratifying on legal sex and age group (adolescents <18 years, adults 18–65 years, and older adults ≥65 years), with model 1 and model 2 refitted within each stratum. Moreover, the potential influence of psychiatric comorbidity was evaluated by re-running model 2 six times, sequentially adding each psychiatric disorder cluster to the covariate set. All statistical procedures were performed in SAS version 9.4 (SAS Institute, Cary, NC, USA); two-tailed p-values <0.05 were considered statistically significant.

### Role of the funding source

The funder of the study (Taiwan National Science and Technology Council) had no role in the study design, data collection, data analysis, data interpretation, writing of the manuscript, or the decision to submit the paper for publication.

## Results

**Fig. 1:**
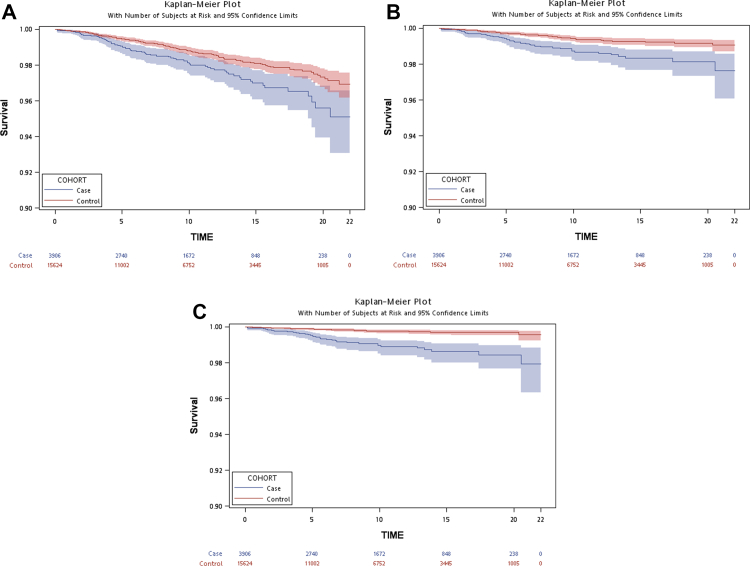
Kaplan–Meier survival curves in transgender persons and matched controls. (A) All-cause; (B) Unnatural causes; (C) Suicide.

**Fig. 2:**
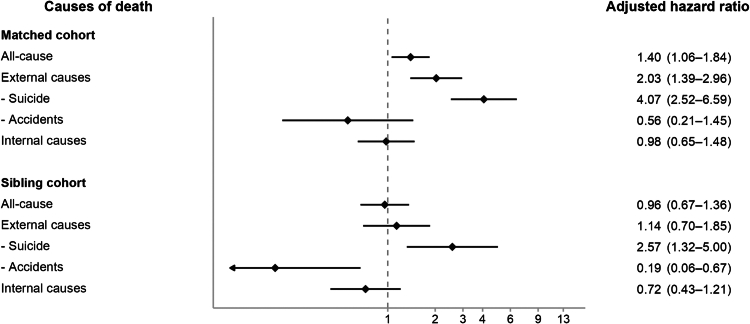
Adjusted hazard ratios with 95% confidence intervals for all-cause and cause-specific mortality in transgender persons vs matched controls and cisgender sibling controls. Models adjust for birth year, legal sex, income quartile, urbanization level, and Charlson Comorbidity Index; sibling-comparison models additionally adjust for birth order. The forest plot displays adjusted hazard ratios with 95% confidence intervals. The horizontal axis is logarithmic, and the vertical dashed line marks the null value (hazard ratio = 1).

Table 1 and Table S2 summarize the demographic and clinical characteristics of the study population. Between 2001 and 2021, we identified 3906 transgender persons and 15,624 controls matched by legal sex and age. The mean age at first recorded GID diagnosis was 24.6 years, and 70.6% of the transgender cohort had a male legal sex. The mean follow-up duration was 9.5 years. Medical comorbidity, as assessed by the CCI, was more common among transgender persons than among controls (CCI 1–2: 54.0% vs 51.5%; CCI >2: 16.1% vs 12.6%). Psychiatric conditions were likewise over-represented in the transgender cohort: neurodevelopmental disorders (10.0% vs 3.2%), psychotic disorders (7.3% vs 1.4%), bipolar disorder (7.3% vs 1.0%), depressive disorders (40.8% vs 5.7%), anxiety disorders (29.4% vs 6.0%), and substance use disorders (2.4% vs 0.7%). Among 3906 transgender persons and 15,624 matched controls, 73 (1.9%) and 183 (1.2%) deaths, respectively, were observed over a mean 9.5-year follow-up, yielding crude mortality rates of 19.6 vs 12.2 per 10,000 person-years. Kaplan–Meier survival curves illustrate all-cause and cause-specific mortality in transgender persons and matched controls (Fig. 1A–C; Figure S2A–B). Both model 1 (crude HR, cHR) and model 2, the multivariable-adjusted model (adjusted HR, aHR), showed significantly higher risk in the transgender cohort for all-cause mortality (cHR, 95% CI = 1.61, 1.23–2.11; aHR, 95% CI = 1.40, 1.06–1.84), deaths from external causes (cHR = 2.25, 1.54–3.29; aHR = 2.03, 1.39–2.96), and suicides (cHR = 4.54, 2.80–7.37; aHR = 4.07, 2.52–6.59). In contrast, neither accident-related deaths (aHR = 0.56, 0.21–1.45) nor internal-cause deaths (aHR = 0.98, 0.65–1.48) differed significantly from controls (Table 2; Fig. 2). Two sensitivity analyses produced virtually identical risk patterns (Table S3).

**Table 1:** Characteristics of all included participants from 2001 to 2021.^a^

Characteristics	Transgender persons (n = 3906)	Matched controls (n = 15,624)	Sibling controls (n = 4765)
**Basic information**			
Age, year	24.6 ± 7.5	24.6 ± 7.5	22.5 ± 8.0
Legal sex, male	2757 (70.6)	11,028 (70.6)	2350 (49.3)
**Personal income level**			
<25% (top)	746 (19.1)	3868 (24.8)	1090 (22.9)
25–50%	858 (22.0)	3772 (24.1)	1051 (22.1)
50–75%	765 (19.6)	3357 (21.5)	901 (18.9)
≥75% (bottom)	1254 (32.1)	3587 (23.0)	1195 (25.1)
Unknown	283 (7.2)	1040 (6.7)	528 (11.1)
**Personal urbanization level**			
Level 1 (urban)	2190 (56.1)	8622 (55.2)	2701 (56.7)
Level 2	1412 (36.1)	5645 (36.1)	1683 (35.3)
Level 3	234 (6.0)	1021 (6.5)	289 (6.1)
Level 4 (rural)	44 (1.1)	145 (0.9)	51 (1.1)
Unknown	26 (0.7)	191 (1.2)	41 (0.9)
**Physical comorbidity**			
0 (Charlson Comorbidity Index)	1165 (29.8)	5607 (35.9)	1708 (35.8)
1–2	2111 (54.0)	8042 (51.5)	2525 (53.0)
>2	630 (16.1)	1975 (12.6)	532 (11.2)
**Psychiatric comorbidity**			
Neurodevelopmental disorders	390 (10.0)	501 (3.2)	224 (4.7)
Psychotic disorders	285 (7.3)	217 (1.4)	79 (1.7)
Bipolar disorders	285 (7.3)	154 (1.0)	94 (2.0)
Depressive disorders	1592 (40.8)	895 (5.7)	465 (9.8)
Anxiety disorders	1150 (29.4)	942 (6.0)	453 (9.5)
Substance use disorders	94 (2.4)	105 (0.7)	29 (0.6)

a Data was expressed as mean (standard deviation) or N (percentage).

**Table 2:** Risk of all-cause and cause-specific mortality in transgender persons and matched controls/cisgender sibling controls.

Characteristics	Cases, event^a^	Cases, mortality rate	Matched controls, event^a^	Matched controls, mortality rate	Crude hazard ratio (model 1)
**Matched cohort**	(n = 3906)		(n = 15,624)		
All-cause	73 (1.9)	19.6	183 (1.2)	12.2	1.61 (1.23–2.11)^b^
External causes	42 (1.1)	11.3	75 (0.5)	5.0	2.25 (1.54–3.29)^b^
Suicide	35 (0.9)	9.4	31 (0.2)	2.1	4.54 (2.80–7.37)^b^
Accidents	5 (0.1)	1.3	34 (0.2)	2.3	0.59 (0.23–1.51)
Internal causes	31 (0.8)	8.3	108 (0.7)	7.2	1.16 (0.78–1.73)
**Sibling cohort**	(n = 3906)		(n = 4765)		
All-cause	73 (1.9)	8.6	64 (1.3)	6.3	1.35 (0.97–1.89)
External causes	42 (1.1)	4.9	33 (0.7)	3.2	1.52 (0.96–2.39)
Suicide	35 (0.9)	4.1	12 (0.3)	1.2	3.45 (1.79–6.65)^b^
Accidents	5 (0.1)	0.6	18 (0.4)	1.8	0.33 (0.12–0.90)^b^
Internal causes	31 (0.8)	3.6	31 (0.7)	3.0	1.18 (0.72–1.95)

a Event was expressed as N (percentage) and mortality rate was expressed as event per 10,000 person-years.

b P values < 0.05.

The sibling cohort comprised 4765 cisgender siblings; 64 deaths occurred during follow-up (Tables 1 and 2). After adjustment for age, legal sex, income, urbanization, CCI, and birth order, the excess risks seen in the primary analysis were not observed for all-cause mortality (aHR = 0.96, 0.67–1.36) or for deaths from external causes (aHR = 1.14, 0.70–1.85). Within external causes, suicide hazard was higher in the transgender cohort (cHR = 3.45, 1.79–6.65; aHR = 2.57, 1.32–5.00), whereas accident-related mortality was lower (cHR = 0.33, 0.12–0.90; aHR = 0.19, 0.06–0.67).

**Fig. 3:**
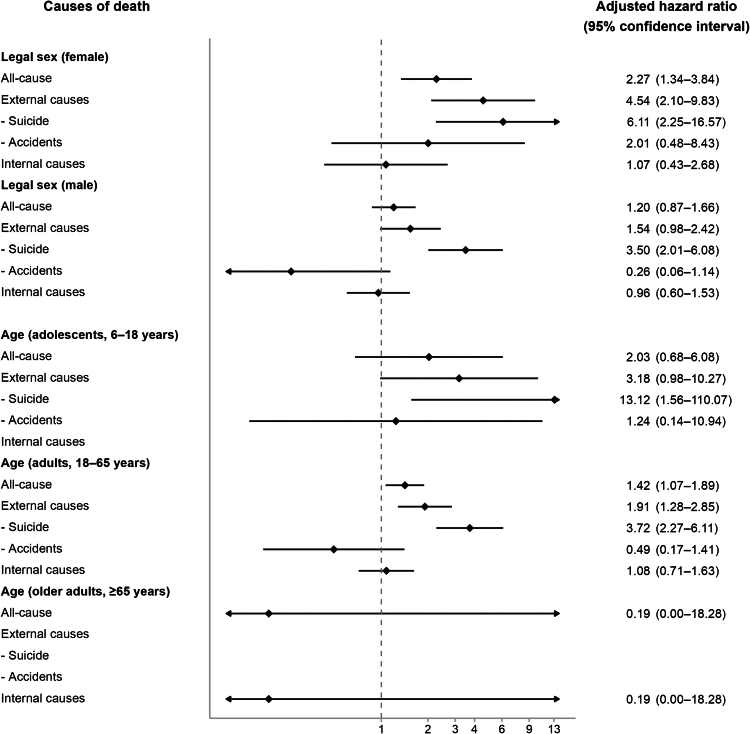
Adjusted hazard ratios with 95% confidence intervals for all-cause and cause-specific mortality in transgender persons vs matched controls, by legal sex or age. Models adjust for birth year, legal sex, income quartile, urbanization level, and Charlson Comorbidity Index. The forest plot displays adjusted hazard ratios with 95% confidence intervals. The horizontal axis is logarithmic, and the vertical dashed line marks the null value (hazard ratio = 1).

Sex-stratified analyses demonstrated a consistently greater excess risk in individuals with a female legal sex than in those with a male legal sex (Table S4; Fig. 3). For all-cause mortality, the aHR was 2.27 (95% CI 1.34–3.84) in females vs 1.20 (0.87–1.66) in males. For deaths from external causes, the aHRs were 4.54 (2.10–9.83) and 1.54 (0.98–2.42), respectively. For suicide, the aHRs were 6.11 (2.25–16.57) and 3.50 (2.01–6.08), respectively. Age-stratified analyses showed a similar gradient: transgender adolescents displayed higher relative hazards than adults for all-cause (aHR, 95% CI = 2.03, 0.68–6.08 vs 1.42, 1.07–1.89), external-cause (3.18, 0.98–10.27 vs 1.91, 1.28–2.85), and suicide mortality (13.12, 1.56–110.07 vs 3.72, 2.27–6.11). In the older-adult stratum, there were only seven transgender persons (one death by internal cause) and 25 matched controls (five deaths by internal causes), and the sample size was too small to perform detailed subgroup analyses (Table S4). Stratification by psychiatric comorbidity produced risk profiles that broadly paralleled the primary analysis, yet effect sizes—and thus statistical significance—varied across disorder clusters (Table S5). For example, suicide mortality remained significantly elevated among transgender persons who also carried neurodevelopmental, psychotic, bipolar, anxiety, or substance use disorders, whereas the association lost significance in those with comorbid depressive disorders (aHR, 95% CI = 1.64, 0.86–3.10).

## Discussion

Over a 22-year observation window, this population-based cohort study tracked 3906 transgender persons and 15,624 matched controls. Relative to controls, the transgender cohort showed a 40% higher adjusted hazard of all-cause mortality, primarily attributable to deaths from external causes (aHR = 2.03), with suicides contributing most markedly (aHR = 4.07). By contrast, the hazard of fatal accidents was lower in the transgender group (aHR = 0.56), although this reduction did not reach statistical significance. These risk patterns were similar in two sensitivity analyses. Furthermore, a sibling-comparison design, which controls for shared genetics and early-life environment, revealed no excess hazard for either all-cause (aHR, 95% CI = 0.96, 0.67–1.36) or external-cause mortality (aHR, 95% CI = 1.14, 0.70–1.85). The suicide hazard remained elevated (aHR = 2.57). Notably, accidental mortality was lower among transgender persons than cisgender siblings (aHR = 0.19). Two stratified analyses revealed that the greatest relative hazards for both all-cause and cause-specific mortality occurred among participants with a female legal sex (by sex stratification) and among adolescents (by age stratification). Analysis using the psychiatric comorbidity model demonstrated broadly similar risk patterns to the primary findings, although the effect sizes for all-cause and cause-specific mortality were attenuated across all comorbidity clusters.

Consistent with earlier nationwide reports from Finland,^28^ Denmark,^9^ the Netherlands,^8^ and Sweden,^7^ our data show that transgender persons face a significantly higher risk of suicide mortality compared with matched controls from the general population. This excess burden of suicide deaths substantially drives the elevated mortality from external causes, which in turn contributes to the overall higher all-cause mortality observed in this population. Three interacting mechanisms may explain this vulnerability. First, chronic psychological distress arising from untreated gender dysphoria engenders persistent emotional pain and depression,^29^ exacerbating feelings of hopelessness and self-injurious ideation.^30^^,^^31^ Second, life-long exposure to bullying, harassment, and physical assault intensifies minority stress, a pathway well-documented to precipitate suicidal behavior.^32^^,^^33^ Third, familial rejection deprives adolescents of essential social support, heightens social isolation, and leaves enduring psychological scars that perpetuate suicide risk into adulthood.^30^ Moreover, we interpret these population-level findings alongside a complementary, though not fully independent, within-family sibling comparison that implicitly controls shared familial factors (e.g., genetics, early-life context). In that design, all-cause and external-cause mortality did not differ from siblings, whereas suicide remained higher and accident mortality lower for transgender persons. We view the analyses as answering different questions: the population comparison estimates real-world disparity; the sibling comparison probes family-shared factors. Agreement on elevated suicide across designs strengthens confidence, while differences for other outcomes suggest contributions of familial confounding and design conditioning to population-level estimates.

Existing evidence on internal-cause mortality in transgender populations is limited and inconsistent. A Dutch national registry study reported excess cardiovascular, lung cancer, and HIV-related deaths among transgender women,^8^ whereas a large U.K. cohort study found no elevation in cancer- or cardiovascular-specific mortality among transgender persons.^34^ Consistent with the latter, our data show no increase in internal-cause mortality, a finding that likely reflects the youthful age structure of our cohort (mean age 25 years), in which cardiometabolic and malignant deaths are uncommon. Moreover, accidental mortality has rarely been analyzed separately; most investigations subsume it under “external causes,” and the few that have isolated accidents—principally registry studies from the Netherlands^8^ and Sweden^7^—have reported non-significant risk elevations. By contrast, we observed no significant reduction in accidental deaths in the matched-pair control analysis and further found a significant 81% risk reduction in the sibling comparison analysis. Two behavioral mechanisms may underlie this pattern. First, structural marginalization restricts access to high-risk pursuits such as contact or competitive sports.^35^ A recent systematic review showed markedly lower sports participation among transgender people owing to discrimination, lack of inclusive facilities, and policy barriers.^36^ Second, pervasive psychological distress and the threat of interpersonal violence foster anticipatory coping, whereby transgender persons consciously avoid outdoor adventures and other contexts perceived as unsafe.^7^ These self-protective adaptations, particularly evident when contrasted with genetically similar cisgender siblings, likely curtail exposure to hazardous situations and thus underpin the pronounced deficit in accident-related mortality observed here.

Sex-stratified models showed that for every endpoint, participants with a female legal sex had higher aHRs than those with a male legal sex, relative to their respective cisgender controls. This disparity probably reflects the lower baseline mortality in the general female population; therefore, increased transgender-related mortality in individuals with female legal sex is proportionally greater when compared with female controls (Table S4). An analogous phenomenon emerged in the age stratum. Although absolute mortality among adolescent transgender persons approached that of adult transgender persons, background mortality in the general adolescent population is extremely low; consequently, the mortality differential between adolescent transgender persons and their matched peers exceeded that observed in adults. These findings underscore the imperative to prioritize early mental-health support and suicide-prevention initiatives for transgender youth. Sequential adjustment for psychiatric comorbidity indicated heterogeneity across the six clusters. Neurodevelopmental disorders altered aHRs minimally, whereas depressive disorders produced the greatest attenuation. Because psychiatric comorbidities were defined without enforcing temporal order relative to the index GID-coded diagnosis, we cannot definitively distinguish confounding from mediation. Neurodevelopmental disorders typically precede the index diagnosis, whereas other disorders (e.g., depressive disorders) may occur before or after. In this study, we adjusted for psychiatric comorbidities in the database; however, prior literature is inconclusive regarding which psychiatric conditions act as confounders vs mediators.^7^^,^^8^^,^^34^^,^^37^ Future work could explicitly map temporal ordering and apply causal frameworks—such as directed acyclic graph-informed covariate selection, time-updated covariates, and formal mediation analyses—to clarify pathways.

In Taiwan, advances in recognition and care for transgender and gender-diverse persons coexist with persistent structural barriers that shape health risk. Legal sex-marker change has historically required two psychiatric opinions and proof of genital surgery.^15^ Anti-discrimination protections remain fragmented, and services are concentrated in metropolitan areas. National Health Insurance does not reimburse gender-affirming surgery and inconsistently covers medications, creating substantial out-of-pocket costs.^6^ Social acceptance is uneven; stigma in schools and workplaces and family rejection are common.^38^ These conditions plausibly intensify psychological distress and help contextualize the excess suicide mortality we observed, particularly among adolescents and those with a female legal sex. Actionable steps follow directly from these findings. Clinically, routine, structured suicide-risk assessment should be embedded at key touchpoints of transgender and gender-diverse care—especially during adolescence—including documentation of ideation, intent, plans, access to means, and past behaviors, followed by safety planning, lethal-means counseling, and rapid linkage to affirming mental-health services.^39^ Primary care and mental health clinicians should receive training in gender-affirming, culturally competent care, with clear referral pathways and warm handoffs. Policy priorities include extending National Health Insurance coverage to evidence-based gender-affirming treatments (hormones and surgeries), expanding access outside major cities, enforcing school anti-bullying provisions, and investing in family-acceptance and peer-support programs. Embedding these measures within health and education systems can translate our epidemiologic findings into measurable public health benefit for transgender and gender-diverse persons in Taiwan.

To our knowledge, this is the largest population-based investigation of transgender persons in Asia. It evaluates all-cause and cause-specific mortality relative to matched population controls, and crucially incorporates a sibling-comparison design, an approach that accounts for shared genetic and early-life environmental factors. However, several limitations warrant consideration. First, lifestyle covariates such as diet, smoking, and occupational exposures were unavailable, limiting adjustment for non-disease determinants of mortality. Second, legal sex was inferred from the earliest sex entry in the NHIRD (available from 2001 onward). Because gender-affirming surgeries performed before this date could not be identified, there may be discrepancies between legal sex and assigned sex at birth, which could affect sex-stratified estimates. Third, cause-specific deaths were infrequent, especially among older adults, reducing statistical power for certain endpoints. Fourth, because a GID diagnosis by a psychiatrist is required for gender-affirming care in Taiwan, most individuals in the transgender cohort had at least one psychiatric diagnosis. This may lead to differential ascertainment compared with cisgender controls, in whom psychiatric conditions may remain undiagnosed, suggesting potential under-adjustment when controlling for comorbidity. Future studies could also examine mortality within subgroups defined by documented psychiatric diagnoses to ensure comparability. Finally, our cohort includes only transgender individuals who sought and received a GID diagnosis, excluding those unwilling to disclose their identity or not seeking gender-affirming care, and thus does not represent the entire community. The study was conducted within Taiwan's healthcare system, which partially represents the study's applicability to populations across the Western Pacific region. However, generalizability to area with markedly different healthcare infrastructures and cultural environments remains uncertain.

### Conclusion

This population-based cohort of 3906 transgender persons showed a 40% excess in all-cause mortality vs matched controls, almost entirely attributable to a two-fold elevation in deaths from unnatural causes—principally a four-fold higher suicide mortality risk—while natural causes were unchanged and accidents fewer. In cisgender sibling comparisons, suicide mortality remained elevated among transgender persons. Individuals with a female legal sex and adolescents bore the greatest relative risks. These findings support policies that prioritize targeted, proactive mental-health interventions for these groups to reduce suicide and improve long-term survival.

## Contributors

Research idea and study design: CWH; data acquisition and interpretation: CAH and CWH; statistical analysis: CWH; manuscript drafting: CWH; manuscript revision: CWH, YCC, LJW, MHC, CSL, YHY, PYC, and CCL. CWH directly accesses and verifies data and has full access to all data. Each author contributed important intellectual content during manuscript drafting or revision and accepts accountability for the overall work by ensuring that questions pertaining to the accuracy or integrity of any portion of the work are appropriately investigated and resolved. CWH had final responsibility for the decision to submit for publication.

## Data sharing statement

The data of the current study would be available to the corresponding author upon reasonable request and approval from the Institutional Review Board and permission from the Taiwan Ministry of Health and Welfare.

## Ethical consideration

The research was conducted ethically in accordance with the World Medical Association Declaration of Helsinki. The Institutional Review Board of Chang Gung Memorial Hospital approved the study protocol and waived the need for informed consent (No.: 202300262B0).

## Declaration of interests

All authors declare no financial interests or potential conflicts of interest regarding the authorship and publication of this article.
