# Supplementary material for: Mortality among transgender persons: a Taiwan matched-population and sibling-comparison cohort study
Source: Lancet Reg Health West Pac. 2025 Oct 18;64:101715. doi: 10.1016/j.lanwpc.2025.101715 (PMC12556306; doi:10.1016/j.lanwpc.2025.101715)
Supplement: Supplementary Figures and Tables [file mmc1.docx]

**Supplementary material**

**Mortality Among Transgender Persons: A Taiwan Matched-Population and Sibling-Comparison Cohort Study**

| **Content** | **Page** |
| --- | --- |
| **Table S1.** The diagnostic codes for transgender and comorbidities | 1 |
| **Table S2.** Comorbidity details of the Charlson Comorbidity Index | 2 |
| **Table S3.** Risk of all-cause and cause-specific mortality in transgender persons and matched controls (sensitivity analyses) | 3 |
| **Table S4.** Risk of all-cause and cause-specific mortality in transgender persons and matched controls, by legal sex or age | 4 |
| **Table S5.** Risk of all-cause and cause-specific mortality between transgender persons and matched controls, by psychiatric comorbidity group | 5 |
| **Figure S1.** Flowchart of the selection process for the cohort study | 6 |
| **Figure S2.** Kaplan-Meier survival curves in transgender persons and matched controls | 7-8 |
| **Figure S3.** Kaplan-Meier survival curves in transgender persons and matched controls, by legal sex or age | 9-10 |

**Table S1.** **The diagnostic codes for transgender and comorbidities**

|  | International Classification of Diseases 9^th^ and 10^th^ |
| --- | --- |
| Transgender | 302.5 (302.50, 302.51, 302.52, 302.53), 302.6, 302.85; F64 (F64.0–F64.9) |
| Neurodevelopmental disorders (intellectual disability)  (autism spectrum disorders)  (attention-deficit/hyperactivity disorder)  (Tourette syndrome and tic disorder) | 317–319; F70–F73, F78, F79  299; F84  314; F90  307.2; F95 |
| Psychotic disorders | 295, 297, 298.1, 298.3, 298.4, 298.8, 298.9; F20, F22-F25, F28, F29 |
| Bipolar disorders | 296.0, 296.1, 296.4–296.7, 296.80, 296.81, 296.89, 301.13; F30, F31, F34.0 |
| Depressive disorders | 296.2, 296.3, 300.4, 311, 625.4; F32, F33, F34.1 |
| Anxiety disorders | 300.3; F42 |
| Substance use disorders | 295, 297, 298.1, 298.3, 298.4, 298.8, 298.9; F20, F22–F25, F28, F29 |

**Table S2. Comorbidity details of the Charlson Comorbidity Index**

| Characteristics | Transgender persons (n = 3906) | Matched controls  (n = 15,624) | Sibling controls  (n = 4765) |
| --- | --- | --- | --- |
| Acute myocardial infarction | 12 (0.3) | 53 (0.3) | 5 (0.1) |
| Congestive heart failure | 38 (1.0) | 152 (1.0) | 48 (1.0) |
| Peripheral vascular disease | 56 (1.4) | 188 (1.2) | 50 (1.0) |
| Cerebral vascular disease | 138 (3.5) | 445 (2.8) | 148 (3.1) |
| Dementia | 11 (0.3) | 13 (0.1) | 3 (0.1) |
| Pulmonary disease | 1754 (44.9) | 6116 (39.1) | 1964 (41.2) |
| Connective tissue disease | 110 (2.8) | 398 (2.5) | 136 (2.9) |
| Peptic ulcer disease | 1261 (32.3) | 4430 (28.4) | 1336 (28.0) |
| Liver disease | 714 (18.3) | 2663 (17.0) | 651 (13.7) |
| Diabetes | 222 (5.7) | 935 (6.0) | 240 (5.0) |
| Diabetes with complications | 37 (0.9) | 204 (1.3) | 39 (0.8) |
| Hemiplegia or paraplegia | 43 (1.1) | 198 (1.3) | 57 (1.2) |
| Renal disease | 114 (2.9) | 444 (2.8) | 97 (2.0) |
| Cancer (also includes lymphoma / leukemia) | 106 (2.7) | 426 (2.7) | 110 (2.3) |
| Metastatic cancer | 18 (0.5) | 92 (0.6) | 23 (0.5) |
| Severe liver disease | 21 (0.5) | 60 (0.4) | 18 (0.4) |
| Human immunodeficiency virus disease | 146 (3.7) | 50 (0.3) | 18 (0.4) |

^1^ Data was expressed as N (percentage).

**Table S3. Risk of all-cause and cause-specific mortality in** **transgender persons and** **matched controls (sensitivity analyses)**

| Characteristics | Cases, event | Cases, mortality rate | Controls, event | Controls, mortality rate | Crude hazard ratio (model 1) | Adjusted hazard ratio (model 2) |
| --- | --- | --- | --- | --- | --- | --- |
| Excluding data after 2020 | (n = 3050) |  | (n = 12,200) |  |  |  |
| All-cause | 56 (1·8) | 21·5 | 119 (1·0) | 11·3 | 1·90 (1·38–2·60)* | 1·65 (1·19–2·28)* |
| External causes | 33 (1·1) | 12·7 | 57 (0·5) | 5·4 | 2·33 (1·52–3·58)* | 2·08 (1·36–3·18)* |
| Suicide | 26 (0·9) | 10·0 | 22 (0·2) | 2·1 | 4·76 (2·70–8·40)* | 4·05 (2·31–7·10)* |
| Accidents | 5 (0·2) | 1·9 | 27 (0·2) | 2·6 | 0·74 (0·29–1·93) | 0·72 (0·27–1·91) |
| Internal causes | 23 (0·8) | 8·8 | 62 (0·5) | 5·9 | 1·50 (0·93–2·42) | 1·28 (0·78–2·10) |
| Excluding missing data | (n = 3604) |  | (n = 14,479) |  |  |  |
| All-cause | 64 (1·8) | 18·2 | 168 (1·2) | 11·8 | 1·55 (1·16–2·07)* | 1·39 (1·04–1·86)* |
| External causes | 34 (0·9) | 9·7 | 69 (0·5) | 4·8 | 2·00 (1·33–3·02)* | 1·82 (1·21–2·74)* |
| Suicide | 28 (0·8) | 8·0 | 30 (0·2) | 2·1 | 3·79 (2·26–6·34)* | 3·45 (2·06–5·77)* |
| Accidents | 4 (0·1) | 1·1 | 31 (0·2) | 2·2 | 0·52 (0·18–1·48) | 0·51 (0·17–1·47) |
| Internal causes | 30 (0·8) | 8·6 | 99 (0·7) | 6·9 | 1·24 (0·82–1·86) | 1·10 (0·72–1·67) |

^1^ Event was expressed as N (percentage) and mortality rate was expressed as event per 10,000 person-years.

^2^ Model 2 was adjusted for all matching variables (birth year, legal sex, income level, urbanization level, and Charlson Comorbidity Index).

**Table S4. Risk of all-cause and cause-specific mortality in transgender** **persons and matched controls****, by legal sex or age**

| Characteristics | Cases, event | Cases, mortality rate | Controls, event | Controls, mortality rate | Crude hazard ratio (model 1) |
| --- | --- | --- | --- | --- | --- |
| Legal sex |  |  |  |  |  |
| Female | (n = 1149) |  | (n = 4596) |  |  |
| All-cause | 21 (1·8) | 20·1 | 35 (0·8) | 8·3 | 2·42 (1·41–4·16)* |
| External causes | 15 (1·3) | 14·3 | 11 (0·2) | 2·6 | 5·50 (2·53–11·97)* |
| Suicide | 11 (1·0) | 10·5 | 6 (0·1) | 1·4 | 7·39 (2·74–19·97)* |
| Accidents | 3 (0·3) | 2·9 | 5 (0·1) | 1·2 | 2·43 (0·58–10·15) |
| Internal causes | 6 (0·5) | 5·7 | 24 (0·5) | 5·7 | 1·01 (0·41–2·47) |
| Male | (n = 2757) |  | (n = 11,028) |  |  |
| All-cause | 52 (1·9) | 19·5 | 148 (1·3) | 13·8 | 1·42 (1·03–1·94)* |
| External causes | 27 (1·0) | 10·1 | 64 (0·6) | 6·0 | 1·70 (1·08–2·66)* |
| Suicide | 24 (0·9) | 9·0 | 25 (0·2) | 2·3 | 3·86 (2·20–6·75)* |
| Accidents | 2 (0·1) | 0·7 | 29 (0·3) | 2·7 | 0·28 (0·07–1·16) |
| Internal causes | 25 (0·9) | 9·4 | 84 (0·8) | 7·8 | 1·20 (0·77–1·88) |
| Age |  |  |  |  |  |
| Adolescents (6–18 years) | (n = 486) |  | (n = 1969) |  |  |
| All-cause | 6 (1·2) | 9·9 | 9 (0·5) | 3·6 | 2·71 (0·97–7·60) |
| External causes | 6 (1·2) | 9·9 | 6 (0·3) | 2·4 | 4·07 (1·31–12·58)* |
| Suicide | 4 (0·8) | 6·6 | 1 (0·1) | 0·4 | 16·29 (1·83–145·08)* |
| Accidents | 1 (0·2) | 1·6 | 3 (0·2) | 1·2 | 1·35 (0·14–13·00) |
| Internal causes^a^ | 0 (0·0) | 0·0 | 3 (0·2) | 1·2 | – |
| Adults (18–65 years) | (n = 3413) |  | (n = 13,630) |  |  |
| All-cause | 66 (1·9) | 21·3 | 169 (1·2) | 13·6 | 1·57 (1·18–2·09)* |
| External causes | 36 (1·1) | 11·6 | 69 (0·5) | 5·5 | 2·10 (1·40–3·13)* |
| Suicide | 31 (0·9) | 10·0 | 30 (0·2) | 2·4 | 4·15 (2·51–6·85)* |
| Accidents | 4 (0·1) | 1·3 | 31 (0·2) | 2·5 | 0·52 (0·18–1·47) |
| Internal causes | 30 (0·9) | 9·7 | 100 (0·7) | 8·0 | 1·21 (0·81–1·82) |
| Older adults (≥65 years) | (n = 7) |  | (n = 25) |  |  |
| All-cause | 1 (14·3) | 210·8 | 5 (20) | 302·0 | 0·70 (0·08–6·00) |
| External causes^a^ | 0 (0·0) | 0·0 | 0 (0·0) | 0·0 | – |
| Suicide^a^ | 0 (0·0) | 0·0 | 0 (0·0) | 0·0 | **–** |
| Accidents^a^ | 0 (0·0) | 0·0 | 0 (0·0) | 0·0 | **–** |
| Internal causes | 1 (14·3) | 210·8 | 5 (20·0) | 302·0 | 0·70 (0·08–6·00) |

^1^ Event was expressed as N (percentage) and mortality rate was expressed as event per 10,000 person-years.

^a^ No events occurred in the transgender persons or the matched controls.

**Table S5. Risk of all-cause and cause-specific mortality between** **transgender persons and matched controls, by psychiatric comorbidity group**

| Characteristics | Neurodevelopmental disorders | Psychotic disorders | Bipolar disorders | Depressive disorders | Anxiety disorders | Substance use disorders |
| --- | --- | --- | --- | --- | --- | --- |
| All-cause | 1·34 (1·02–1·77)* | 1·11 (0·83–1·49) | 1·09 (0·80–1·47) | 1·00 (0·74–1·36) | 1·20 (0·89–1·61) | 1·24 (0·94–1·65) |
| External causes | 1·90 (1·30–2·78)* | 1·50 (0·99–2·26) | 1·54 (1·01–2·33)* | 1·00 (0·63–1·59) | 1·41 (0·91–2·17) | 1·72 (1·15–2·56)* |
| Suicide | 3·84 (2·37–6·22)* | 3·03 (1·81–5·09)* | 2·83 (1·61–4·99)* | 1·64 (0·86–3·10) | 2·83 (1·63–4·90)* | 3·30 (1·96–5·56)* |
| Accidents | 0·50 (0·19–1·33) | 0·40 (0·14–1·16) | 0·54 (0·21–1·38) | 0·33 (0·11–1·01) | 0·38 (0·12–1·15) | 0·49 (0·18–1·34) |
| Internal causes | 0·96 (0·64–1·45) | 0·83 (0·54–1·28) | 0·78 (0·50–1·22) | 0·96 (0·63–1·46) | 1·00 (0·66–1·52) | 0·91 (0·60–1·37) |

^1^ Data was expressed as adjusted hazard ratio with 95% confidence intervals.

^2^ Adjusted for all matching variables (birth year, legal sex, income level, urbanization level, and Charlson Comorbidity Index).

**Figure S1.** **Flowchart of the selection process for the cohort study**

**
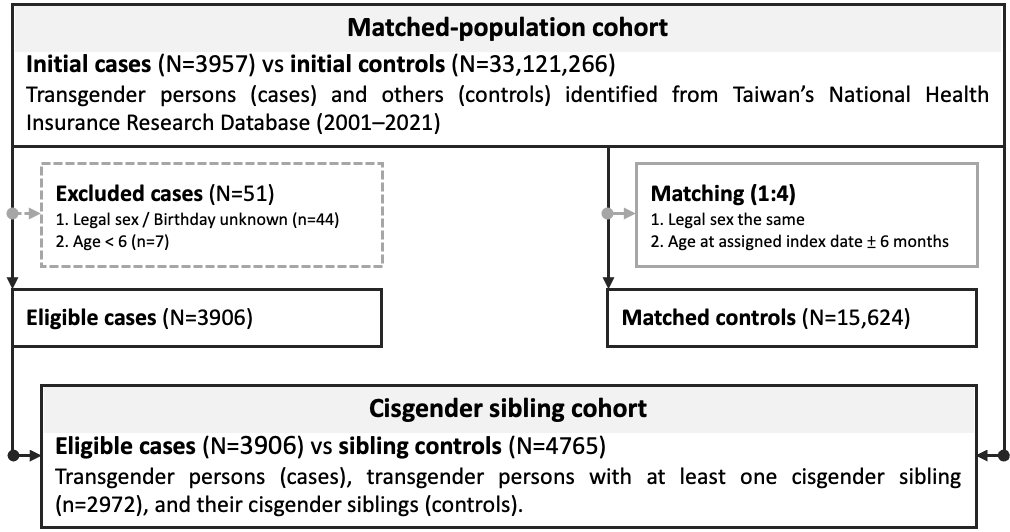
**

**Figure S2. Kaplan-Meier survival curves in** **transgender persons and matched controls**

**A) Accidents**

**
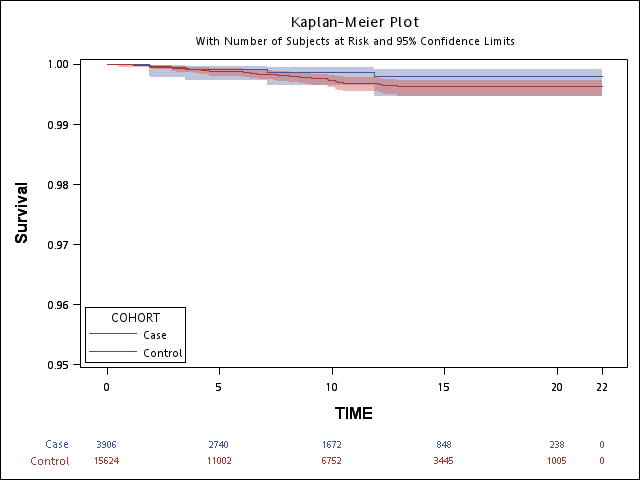
**

**B) Natural causes**

**
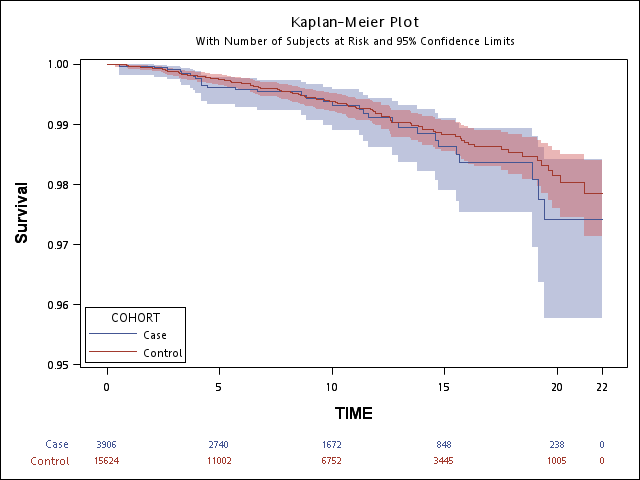
**

**Figure S3. Kaplan-Meier survival curves in transgender persons and matched controls, by** **legal sex or** **age**

**A) All-cause for** **legal sex**

**
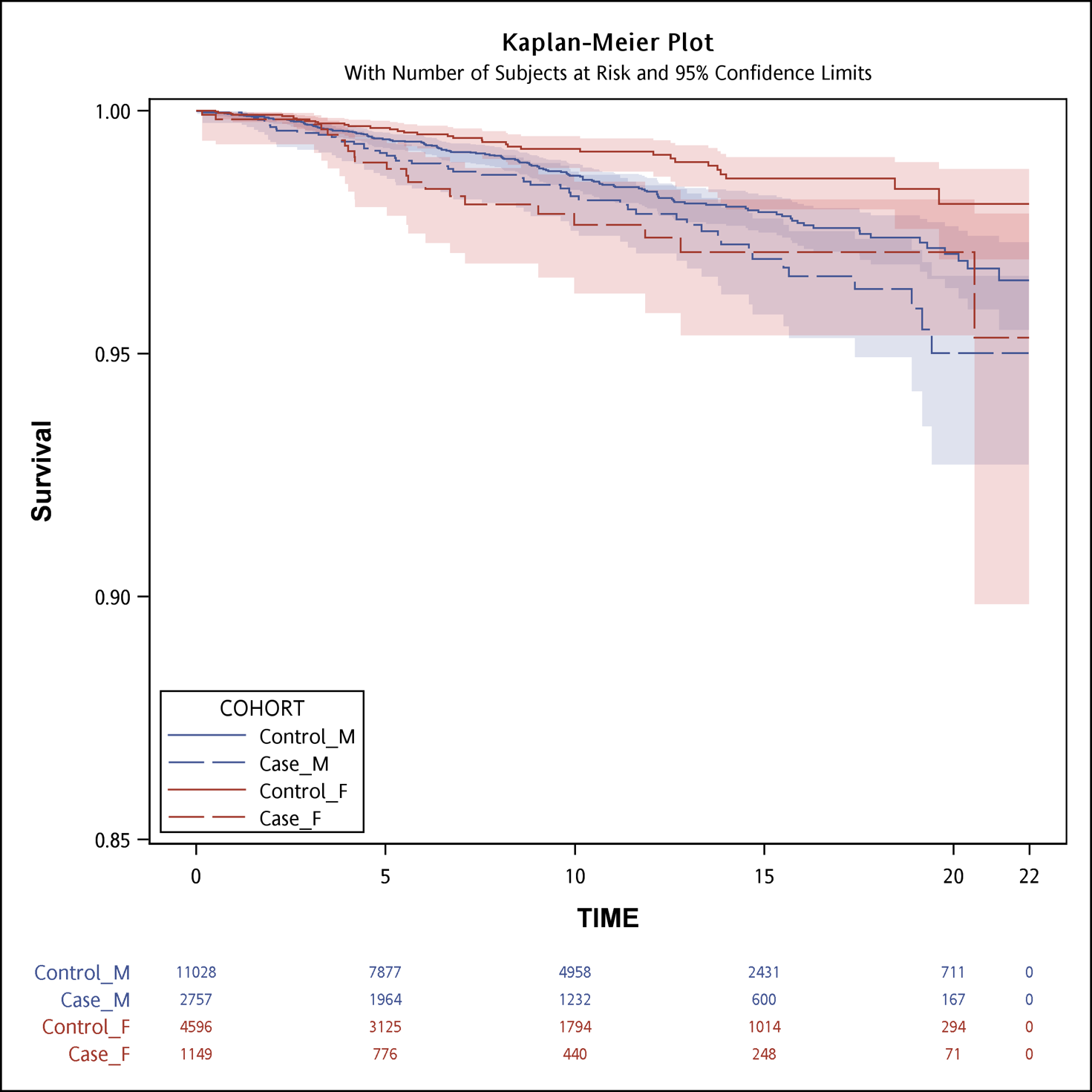
**

M = legal male sex; F = legal female sex

**B) All-cause for age**

**
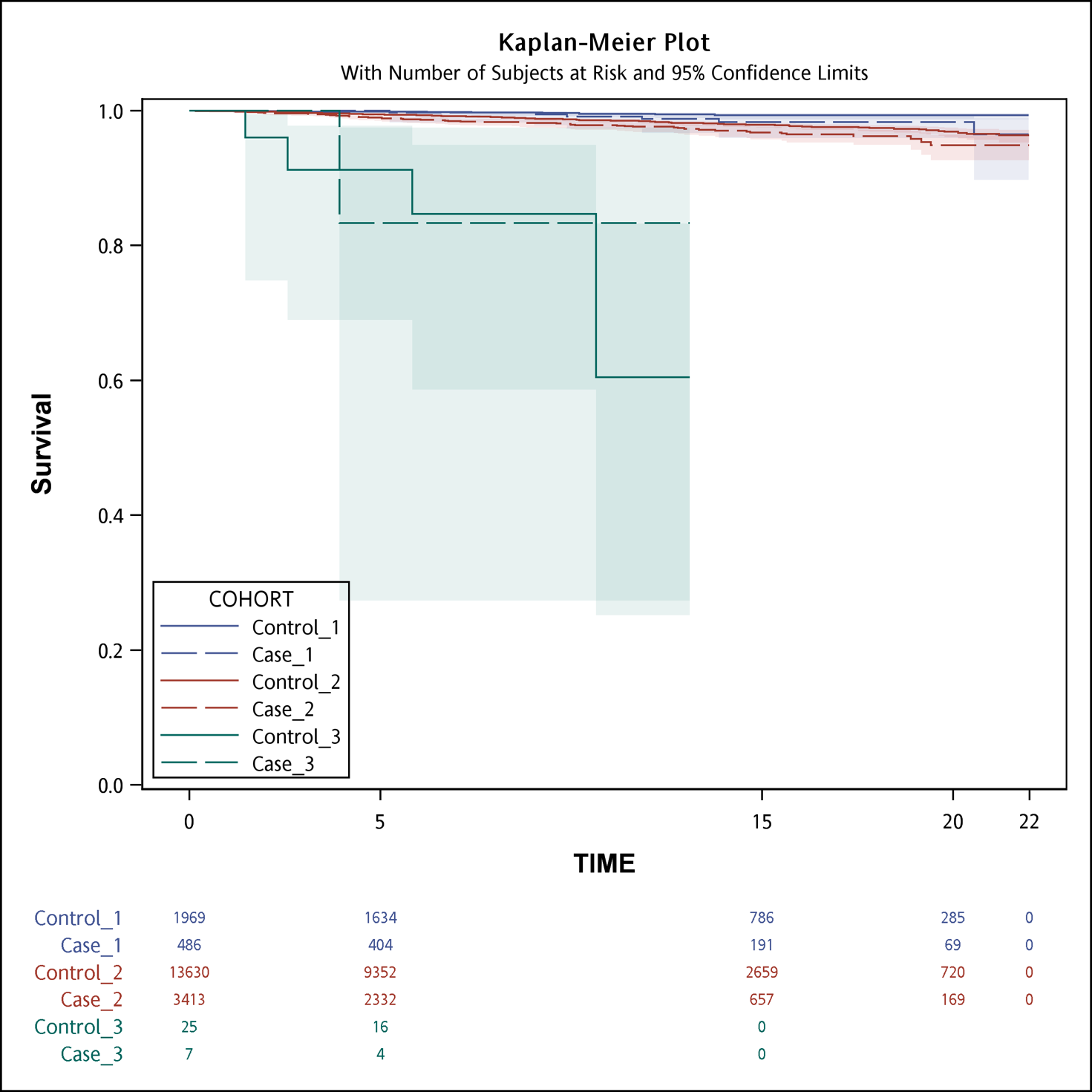
**

1 = adolescents; 2 = adults; 3 = older adults
